# Cyclosporine Dissolution Test from a Lipid Dosage Form: Next Step Towards the Establishment of Release Method for Solid Lipid Microparticles

**DOI:** 10.3390/pharmaceutics17081030

**Published:** 2025-08-08

**Authors:** Eliza Wolska, Patrycja Dudek, Małgorzata Sznitowska

**Affiliations:** 1 Department of Pharmaceutical Technology, Medical University of Gdansk, Hallera 107, 80-416 Gdansk, Poland; 2 Student Chapter of the International Society of Pharmaceutical Engineering (ISPE), Hallera 107, 80-416 Gdansk, Poland

**Keywords:** cyclosporine, solid lipid microparticles, microspheres, release study, dissolution study

## Abstract

**Background:** The release study is a standard tool for the development, evaluation, and control of dosage forms. In the case of traditional drug delivery systems, it is conducted in accordance with the established principles available in the European and American Pharmacopoeias or guidelines proposed by registration agencies. The problem is the study of modern carriers, not yet described in compendia, which require adjustments to traditionally used methods. **Objectives:** The present study focuses on developing an optimal method for testing the release of cyclosporine (Cs, 0.5–4%) incorporated in solid lipid microparticles (SLM) dispersions (10%) intended for administration in the form of eye drops. This is a multicompartment lipid carrier that provides prolonged release of the active substance. **Methods:** Three methods of testing the release were compared: the dialysis bag method, the horizontal cells technique, and a method without a membrane. **Results:** During the analyses, the proper membrane was selected and the effect of the lysozyme enzyme on the release profile was analyzed. The effect of the composition of the acceptor fluid on the obtained results was also assessed. In the model without a membrane, up to 60% of the Cs was released within 30 min due to the *burst effect*. In horizontal chambers, no formulation released more than 14% of the Cs over 96 h, while at the same time, 60–70% of the Cs was released from the dialysis bag. **Conclusions:** Based on the obtained results, the dialysis bag method was selected to study the release of Cs from SLM without the need to use multicomponent artificial tear fluid as an acceptor medium.

## 1. Introduction

Multicompartment and lipid dosage forms, such as liposomes [1], solid lipid nanoparticles (SLN), nanostructured lipid carriers (NLC) [2], solid lipid microparticles (SLM) [3], self-emulsifying drug delivery systems [4], or emulsions [5], offer various advantages over traditional carriers. The lipophilic nature of the carriers makes them more suitable for drug substances with hydrophobic properties and very poor water solubility [1,4]. This is expected to enhance drug loading capacity as well as the bioavailability and stability of loaded drugs [2,6,7,8]. The utilization of particularly liposomes (although some attempts have also been made with SLN and NLC) as a targeted drug delivery system has received considerable attention [2,6,7]. In turn, the continuing interest in the SLM carrier results from its ability to provide prolonged release of active substances [9] and their protection, while maintaining good in vivo tolerance [10].

SLM as a dosage form are characterized by very good physical and chemical stability, and this is also the case in the form of liquid dispersions. The lipid nature of SLM significantly reduces the risk of long-term accumulation in the body, which places this type of carrier in the group of biodegradable carriers [11]. Apart from the numerous advantages mentioned above, SLM dispersion is studied primarily in order to obtain prolonged release. At the time of our previous studies, SLM with cyclosporine A (Cs) suitable for ocular administration had already been developed [12]. Therefore, Cs was selected as a model drug substance. It is practically insoluble in water, with a molecular weight of 1202 daltons and a log P of 2.92 [13,14].

All the properties of lipid dosage forms affect the drug substance and can improve the effectiveness of the applied pharmacotherapy. The release of the drug substance from the carrier is the first and basic condition for the absorption and action of the active substance in the desired place in the body. Lipid dosage forms are also being introduced to provide prolonged release, although this effect is not easily achieved. Another challenge is designing a release study and appropriate in vitro methodology that allows for the development of a formulation with the expected properties.

A release study, also called a dissolution study, is one of the most important analytical tools necessary for drug product development. It is performed for various purposes: to optimize dosage forms, to determine the influence of product variables and production methods on the drug formulations, to perform routine monitoring of quality control, and to optimize an in vitro/in vivo correlation [15,16]. There are many methods described in pharmacopoeias with strictly defined test parameters and required results. Usually, such recommendations refer to traditional, commonly used dosage forms. However, there are no such indications and solutions dedicated to unconventional and modern carriers such as multicompartment lipid carriers, including SLM. Therefore, the continuous development of new release testing models and modification of already used methods is obligatory.

This work focuses on the selection of a release method and appropriate parameters for the assessment of Cs release from an aqueous SLM dispersion intended for administration in the form of eye drops. The aim of the work was to compare different analytical approaches in order to indicate the differences and to prevent misinterpretation of results determined more by the analytical procedure than by the properties of the dosage form. The use of different acceptor fluids or enzyme was intended to achieve in vitro conditions close to physiological ones, which allowed us to obtain results that most closely reflect the behavior of the drug form in vivo at the site of application.

## 2. Materials and Methods

### 2.1. Materials

Cyclosporine A (Cs) was purchased from LC Laboratories (Boston, MA, USA), Compritol 888 ATO (glyceryl behenate) was obtained from Gattefossé (Saint-Priest, France), and polysorbate (Tween 80) and citric acid from Sigma-Aldrich (St. Louis, MO, USA). Lysozyme (LS) from chicken egg white was obtained from Pol-Aura (Olsztyn, Poland). Sodium lauryl sulfate (SLS), sodium chloride, ammonium chloride, potassium chloride, calcium chloride, and magnesium chloride were sourced from POCH (Gliwice, Poland), while sodium bicarbonate was purchased from Chempur (Piekary Śląskie, Poland), urea from Fagron (Cracow, Poland), and lactic acid from B&K (Bytom, Poland). All other chemicals used were of analytical reagent grade. A Milli-Q system (Millipore, Milford, MA, USA) was employed for obtaining high-quality water.

### 2.2. Preparation of SLM Dispersions

The composition of the tested microparticle dispersions was as follows: 10% (*w*/*w*) solid lipid (Compritol), 5% (*w*/*w*) polysorbate, and Cs was used at a concentration of 0.5%, 2%, or 4% (*w*/*w*). SLM formulations were prepared by the previously described hot emulsification method [12]. The emulsification process was carried out using an Ultra-Turrax (T25 Janke-Kunkel, IKA Labortechnik, Staufen, Germany) high-shear mixer for 5 min at 8000 rpm, maintaining a temperature of 80 °C. Before homogenization the active substance was dissolved in molten lipid. The final SLM dispersions were stored in a refrigerator.

### 2.3. Characteristics of the Microspheres’ Properties

#### 2.3.1. Distribution of Cs in SLM Dispersions

The tested SLM formulations were evaluated primarily in terms of the distribution of Cs between the individual dispersion phases: the aqueous phase, the interphase, and the lipid matrix. The analysis was carried out according to a previously developed and characterized procedure [17].

#### 2.3.2. Particle Size Analysis

The particle size was assessed by the laser diffraction method (Beckman-Coulter LS 13 320, Indianapolis, IN, USA) using a Universal Liquid Module. The SLM dispersion was added to the sample cell until the correct obscuration parameter was obtained. The particle size distribution was characterized, in accordance with the recommendations of the pharmacopoeial monograph (European Pharmacopoeia), according to the d_50_ and d_90_ parameters and the mean value.

#### 2.3.3. Scanning Electron Microscopy

The shape, appearance, and morphology of the lipid microspheres were assessed by scanning electron microscopy (SEM) using the Phenom Pro (Phenom World Thermo Fisher, Eindhoven, The Netherlands) after gold sputtering. An acceleration voltage of 15 kV was applied to record images at a magnification of 25,000×.

### 2.4. Drug Release Study

Three different methods were used to study Cs release from SLM: two using a dialysis membrane and the third one without a membrane. In the presented studies, they were marked with the following abbreviations: DBDT–dialysis bag diffusion technique, DCh–diffusion chamber (Side-Bi-Side), MFS–membrane-free system.

Regardless of the method, the temperature was maintained at a constant 37 °C throughout the study. For the DBDT and DCh methods, at the predetermined time points, 1.0 mL of the release medium was withdrawn and replaced with an equal quantity of the same fresh acceptor medium. The Cs concentration in the fluid was determined by HPLC after appropriate dilution with methanol. The total amount of released Cs is most often expressed as a percentage of the total drug content or, for comparison of membrane methods, as the amount of substance diffusing through the membrane surface (in µg/cm^2^).

#### 2.4.1. Dialysis Bag Diffusion Technique (DBDT)

In the DBDT method, the SLM dispersion (1.0 g) was placed in a dialysis bag, which was immersed in the acceptor fluid (70 mL) in a beaker and shaken in a mechanical bath (150 cycles/min). In selected experiments, in order to ensure direct contact between the lysozyme enzyme (LS) and the lipid microparticles, the dispersion in the dialysis bag was diluted (4:1)with dissolved LS. The lysozyme concentration determined on the basis of literature data [18] was 1.4 mg/mL. The diffusion surface area was maintained (at 6.4 cm^2^) by using membranes with the same length and width for all the tests.

#### 2.4.2. Diffusion Chamber (DCh)

For the DCh method 1.0 g of SLM dispersion was placed in a 1 mL donor chamber separated from a 5 mL acceptor chamber (Side-Bi-Side chambers, PermeGear, Hellertown, PA, USA) by a membrane. Both the dispersion and the acceptor fluid in the chambers were magnetically mixed. The diffusion surface area in this system was 0.66 cm^2^.

For both membrane methods (DMDT, DCh), the same cellulose membrane with a molecular weight cut-off (MWCO) of 1000 KDa (Repligen Corporation, Anaheim, CA, USA) was used. In the first stage of the studies, a membrane with a MWCO of 14 KDa (Sigma-Aldrich, St. Louis, MO, USA) was also applied.

#### 2.4.3. Membrane-Free System (MFS)

For the MFS method, an appropriate amount of SLM dispersion (50–200 mg, depending on the Cs concentration) was diluted directly in the acceptor fluid (5 mL) and then mixed in a mechanical shaking bath (150 cycles/min). This procedure required the preparation of a separate sample for each time point, at which the diluted dispersion was filtered through a 0.2 µm filter (Alchem, Torun, Poland).

### 2.5. Composition of Fluids Used in Release Studies

Artificial tear fluid with 0.5% SLS (AT/SLS) or 0.5% SLS solution with sodium chloride (NaCl/SLS) was used as the acceptor fluid in the Cs release study to provide *sink* conditions throughout the experiments. In some experiments, the same fluids were used with the addition of the LS at a concentration of 1.4 mg/mL. The composition of the artificial tear fluid (AT), based on the available data [18,19,20], was as follows: NaCl (99 mmol/L), KCl (20 mmol/L), NH_4_Cl (3 mmol/L), MgCl_2_ × 6H_2_O (0.8 mmol/L), anhydrous CaCl_2_ (0.8 mmol/L), NaHCO_3_ (28 mmol/L), lactic acid (3.4 mmol/L), citric acid (0.04 mmol/L), and urea (5.5 mmol/L).

### 2.6. Solubility Studies of Cs

Before the release tests, the solubility of Cs was determined in water, 0.9% sodium chloride solution, artificial tear fluid, and in the same solutions but with the addition of 0.5% SLS and LS (1.4 mg/mL). The solubility test was performed three times according to the procedure described previously [9].

### 2.7. HPLC Analysis

The concentration of Cs in the tested samples was analyzed by reverse-phase high-performance liquid chromatography (RP-HPLC) using the Prominence LC-2030C 3D apparatus (Shimadzu Corporation, Kioto, Japan). The mobile phase consisted of acetonitrile/water/t-butylmethylether/ortophosphoric acid (520:430:50:1, *v*/*v*) with a flow rate of 2 mL/min. A 250 × 4 mm column (LiChrospher 100 RP-18, Merck KGaA, Darmstadt, Germany) was used, the temperature of the analysis was 80 °C, and the detection wavelength of the UV detector was 210 nm.

### 2.8. Statistical Analysis

All experiments were performed at least three times, and the results are presented as the mean value with the standard deviation (±SD). The one-way analysis of variance (ANOVA) statistical method was used to compare and evaluate the obtained results. Differences were considered significant when *p* ≤ 0.05.

## 3. Results and Discussion

### 3.1. Characterization of Lipid Microparticles

**Figure 1:**
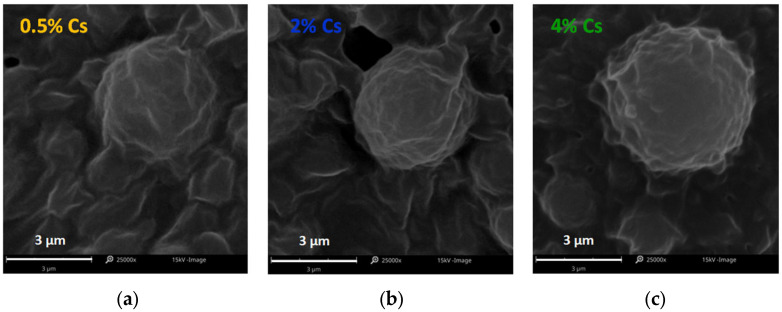
SEM images of SLM with (**a**) 0.5%, (**b**) 2%, and (**c**) 4% Cs.

All prepared and tested SLM with Cs were in the form of liquid dispersions with a milky color. The average size of the obtained lipid microparticles was about 3–4 µm in all formulations (Table 1) and increased slightly with the increase in the concentration of Cs (from 0.5 to 4%) in SLM. All microspheres were spherical in shape (Figure 1). Such physical properties of the dispersions guarantee their ease of application in the form of eye drops. Both the consistency and the size of the particles justify good tolerance after application to the conjunctival sac [21].

A key feature of solid microparticles is the distribution of the active substance between the individual dispersion phases [17]. As shown in Table 1, Cs was distributed both in the lipid matrix and in the interphase (i.e., more at the surface). The partitioning between these two phases depended mainly on the Cs concentration in the formulation. At a low concentration (0.5% Cs), the ratio was 1:1, while at 2% or 4% Cs, it changed to 1:2 with a predominance in the interphase. However, this surface localization effect was not clearly visible in SEM observations (Figure 1). The fraction of the drug substance in the aqueous phase was practically negligible (<0.4%), which resulted from the properties of Cs (which is practically insoluble in water [22]).

### 3.2. Solubility Studies of Cs

**Figure 2:**
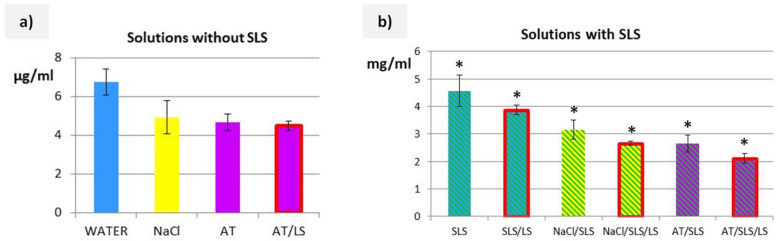
The solubility of Cs in acceptor media (**a**) without SLS and (**b**) with SLS (mean ± SD, * statistically significant difference between solutions of the same composition with and without SLS, i.e., (**a**) vs. (**b**). Solutions with the addition of lysozyme are marked with a red border.

Figure 2 presents the results of Cs solubility tests in solutions with and without 0.5% SLS. As we observed in previous studies on microspheres with indomethacin [9], the use of physiological components (which act on the dosage form at the site of its application) in the composition of the acceptor fluid is justified. Therefore, for the SLM dispersion with potential use as eye drops, 0.9% sodium chloride solution or artificial tear fluid (AT) were considered as solvents that could be the acceptor fluid. Unfortunately, in all these solutions, similarly to water, the solubility of Cs did not exceed 10 µg/mL (Figure 2a). Therefore, SLS at a concentration of 0.5% [23] was used as an additive. In all fluids with SLS, the solubility of Cs exceeded 2 mg/mL (Figure 2b). This means an approximately 500-fold increase compared to the solubility of Cs in the same solutions but without SLS. In practice, only such solubility allows for the design of a release study with *sink* conditions. Interestingly, both in the presence of SLS (Figure 2b) and without it (Figure 2a), the addition of other substances reduced the solubility of Cs. The introduction of the lysozyme enzyme (LS) did not affect the result of the solubility test, regardless of the solution composition.

### 3.3. Influence of Membrane Properties on the Release Profile

In the first stage, Cs release from SLM was carried out using the dialysis bag method. Dialysis membrane techniques are used in studies of the release of various dosage forms [15]. In the previous article, we have already presented a classification of release testing methods for SLM [10]. We have already used this method in our studies [9,23]. In the case of multicompartment carriers such as SLM, their great advantage is the separation of the tested sample from the acceptor medium, which results in the retention of the sample in the system and removes the need to filter each collected portion of the acceptor fluid. Therefore, the dialysis bag method is particularly suitable for all multicompartment dosage forms, not only SLM formulations. One of the basic challenges related to the utilization of this technique is the selection of the dialysis membrane, or more precisely, its parameter described by the abbreviation MWCO (molecular weight cut-off).

**Figure 3:**
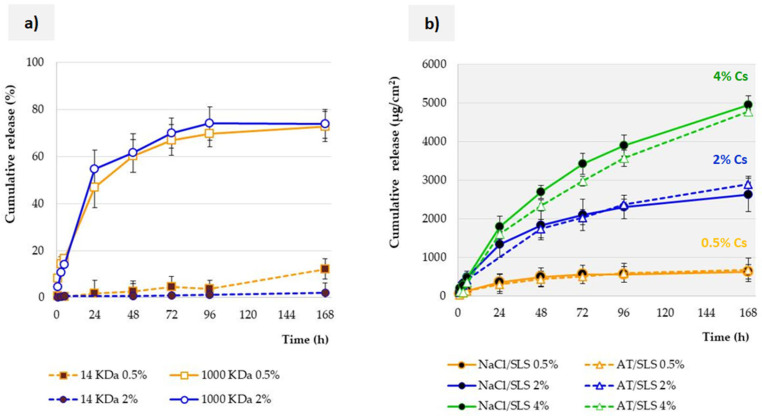
SLM-Cs release profiles (*n* = 3) from dialysis bag: influence of (**a**) membrane MWCO value (14 or 1000 KDa) and (**b**) acceptor fluid composition (NaCl with SLS or artificial tears with SLS).

Figure 3a shows the obtained Cs release profiles from lipid microspheres with an active substance concentration of 0.5% or 2% to the SLS solution (0.5%, with the addition of NaCl). The study was conducted using a dialysis membrane made of regenerated cellulose with an MWCO of 14,000 or 1,000,000 daltons. The obtained results indicate that in the case of the 14 KDa membrane, almost no release occurs. The same comparison was carried out using other acceptor fluids, such as AT or NaCl/SLS with addition of lysozyme. In each case, the same effect was obtained, as shown in Figure 3a.

Cyclosporine, as a polypeptide with a molecular weight of 1202 g/mol, presents lipophilic properties [13,24]. According to the manufacturer’s declaration, the membrane is characterized by very low protein absorption. Moreover, both membranes are made of the same material. Therefore, none of the above reasons is the cause of the observed differences.

According to the idea of the study, the membrane should not restrict permeation of released and dissolved molecules from the donor compartment to the acceptor compartment. Otherwise, this would be a release-limiting factor. As we discussed earlier [23], it is difficult to find precise guidelines on how to select the MWCO. Among the recommendations, there are statements that the MWCO should be adequately large to allow drug transport [15,25]. In the filtration process, it is recommended to choose an MWCO that is 3 to 6 times smaller than the molecular weight of the molecule being retained [26]. Bearing in mind that MWCO describes membrane pore size measured in angstrom (Å) units but also the smallest average molecular mass of a molecule that fails to diffuse across the dialysis membrane [27], it seems reasonable that a 14 KDa membrane will be suitable for a Cs molecule with a mass of 1202 Da. However, the presented results contradict these expectations. The reasons are most likely to be found in the specific properties of Cs as a molecule (not only mass, but also shape and geometry) [28,29]. Some reports indicate that linear molecules can pass more easily through a given pore size (MWCO) compared to a globular molecule of the same molecular weight [30]. Therefore, to achieve free Cs permeation, a much higher MWCO is required (Figure 3a). The recommendation that the MWCO should be high enough to allow the drug to diffuse freely but low enough to prevent the drug carrier (e.g., particles) from passing through is therefore reasonable but imprecise. Developing a universal model that allows the MWCO of a membrane to match the tested active molecule will require taking into account not only its molecular weight but also its spatial structure. When testing new molecules, solutions can be found in spatial modeling or experimental selection. The 1000 KDa membrane selected in our experiment ensured that the carrier was retained in the dialysis bag throughout the study, while preventing release inhibition due to hindered penetration of active substance molecules through the membrane pores.

Since the conducted studies were aimed at comparing different parameters and techniques and their influence on the obtained results, the presented studies were conducted for 96 h, and sometimes even 168 h (as shown in Figure 3), even though the tested SLM dispersion with Cs was intended for ocular administration.

### 3.4. Influence of Acceptor Fluid Composition on the Release Profile

In the conducted studies, two basic types of acceptor fluids were used: a 0.5% SLS solution with the addition of sodium chloride and a multicomponent artificial tear fluid. According to the definition in the European Pharmacopoeia, *sink* conditions are defined as a volume of dissolution medium that is at least three to ten times greater than the saturation volume [16]. This means that the concentration of the active substance should be less than 30% of its solubility limit. Otherwise, the course of the release process may be disturbed, because the release rate, being limited by solubility, will decrease. As such, the obtained release profile will not reflect the properties of the dosage form. In addition, it is necessary to consider the processes occurring in vivo, such as absorption, metabolism, or elimination, which naturally reduce the concentration at the site of administration. These effects are not present in in vitro studies, as already noted by Siepmann et al. [31].

It was found that none of the solutions without SLS was suitable for the release study due to the solubility of Cs being too low (Figure 2). Only the introduction of a surfactant (SLS at a concentration of 0.5%) to the acceptor fluid allowed for a sufficient increase in the solubility of Cs (Figure 2b). According to the guidelines of the EMA and FDA registration agencies [32,33], *sink* conditions should be achieved, although they are not obligatory. In the case of testing substances that are poorly soluble in water, the release fluid is modified using low concentrations of surfactants, while it is advisable to avoid organic solvents. The same is also stated in the guidelines included in the European Pharmacopoeia (5.17.1). Both fluids proposed in our studies, 0.9% NaCl solution and AT (both with SLS), guaranteed *sink* conditions.

The next stage focused on the influence of the remaining components of the acceptor fluid on the release process. When comparing the release profiles of Cs from SLM, apart from single points on the release curve (Figure 3b), no statistically significant differences were found between the release into AT and into isotonic sodium chloride solution. The same results were obtained regardless of the Cs concentration in SLM (0.5–4%). Interestingly, these results, in contrast to our studies of SLM with indomethacin [9], do not confirm the beneficial effect of other components physiologically present at the application site in the course of the release process. This fact should be explained by the specific properties of the tested active substances. Although both Cs and indomethacin are poorly soluble in water, the addition of components such as sodium and citrate ions or urea increased the solubility of indomethacin [9], while they had no significant impact for the polypeptide Cs. From a practical point of view, this is an advantage, as it allows the use of NaCl/SLS as an acceptor fluid in Cs release studies, instead of multicomponent AT/SLS. In this system, sodium chloride increases the osmotic pressure of the acceptor fluid to a similar level as in the tested formulation, which prevents uncontrolled water flow into the bag (according to the concentration gradient). The driving force for the permeation of the released active substance from the donor to the acceptor compartment is the difference in concentrations on both sides of the membrane. On the other hand, such results indicate the different behavior of sparingly soluble active substances. Providing general principles requires testing a larger number of similar substances. In the meantime, when testing a new compound, it is advisable to conduct preliminary analyses (solubility, release), preceding the actual release studies, in order to confirm or exclude the influence of the remaining components of the acceptor fluid on the release profile.

### 3.5. Effect of the Lysozyme Enzyme on the Release Profile

The subject of separate considerations was the influence of the lysozyme enzyme on the release profile. Firstly, the tested SLM dispersion is intended for use in the form of eye drops. Secondly, the degradation of the lipid components of the microparticle matrix occurs under the influence of enzymes [34]. The main enzymes responsible for the degradation of lipid components are lipases, but the effects of other enzymes present in the tear fluid, such as lysozyme, are also studied [35]. Although LS is not a typical lipolytic enzyme, the role of this enzyme in the release of the active substance was demonstrated in the SLM studies with indomethacin [9].

**Figure 4:**
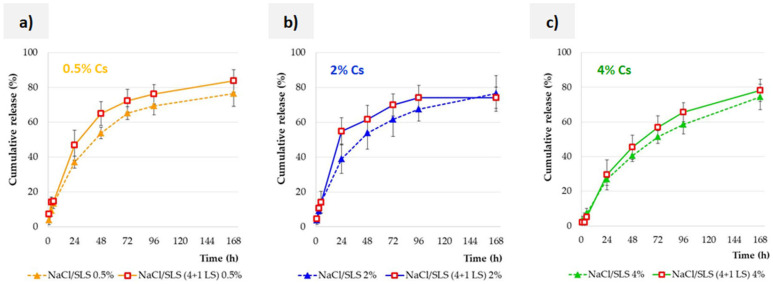
The effect of the LS on the release of Cs from SLM (*n* = 3) with different concentrations of the drug substance: (**a**) 0.5%, (**b**) 2%, and (**c**) 4%. Profiles obtained in the presence of lysozyme are marked with red marker borders and a solid line.

The results of the studies conducted with Cs-loaded SLM are presented in Figure 4. In order to enable direct interaction of the enzyme with lipid microparticles, the enzyme was introduced directly into the dialysis bag (the dispersion was diluted 4:1). The addition of the enzyme had a slight effect on the release rate, regardless of the concentration of Cs in the tested formulation (0.5–4%, Figure 4a–c). Finally, however, the amount of active substance released was the same in the study with and without enzyme (this effect was obtained after about 96 h). Moreover, no further release was observed at a later time. This fact should undoubtedly be connected with the way in which Cs is distributed in lipid microspheres.

As indicated by the distribution results (Table 1), half (0.5% Cs) or two thirds (2%, 4% Cs) of the substance is located on the surface in the interphase, while a smaller part is incorporated in different zones of the lipid matrix. The gradual release of about 80% Cs is definitely more than the fraction of Cs located in the interphase. This confirms the effect observed in our previous studies of SLM with indomethacin [9], in which it was even possible to release 100% of the substance without complete degradation of the lipid matrix. The initial release of the substance located on the surface allows for contact between the subsurface layers of microparticles and the components of the solution, including LS, and further release of the active substance located deeper and deeper. In the case of indomethacin used at a lower concentration (0.2%) and distributed mainly in external layers of microparticles, this effect was sufficient for complete release. According to the distribution results, Cs is incorporated more effectively in lipid matrices than indomethacin [17]. For this reason, a maximum release of 80% of Cs was observed, whereas indomethacin was able to be released completely. Without degradation of the SLM matrix (too short time, no lipases), about 20% of Cs was not released (Figure 4).

The same experiment as described above was carried out using AT/SLS instead of NaCl/SLS as the acceptor fluid. In each case, identical results were obtained. Such results also confirm the lack of influence of other AT components on SLM with Cs, as already described in Section 3.4.

### 3.6. Influence of the Test Method on the Release Profile

**Figure 5:**
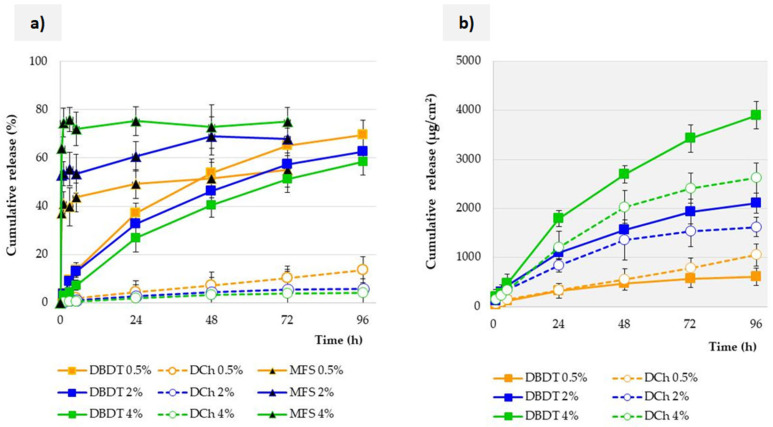
Influence of (**a**) the test method on the Cs release profile from SLM and (**b**) Cs permeation through a 1000 KDa membrane depending on the test model (DBDT: dialysis bag diffusion technique or DCh: diffusion chamber). NaCl/SLS was used as the acceptor fluid (*n* = 3).

As a summary of all the results, the justification for choosing the bag dialysis technique is here presented. Three different methods, with membranes (DBDT and DCh) or without (MFS), were compared, as described in Section 2.4. In all methods, SLM were tested with three different Cs concentrations (0.5–4%). As indicated by the results presented in Figure 5, the release profiles obtained with the three different test methods differ statistically significantly, regardless of the Cs concentration.

The most drastic difference in the obtained results occurs between the study models with and without a membrane. This is related to the occurrence of the *burst effect* in the MFS model, which is manifested by the release of up to 60% of Cs within the initial 30 min. This may be caused not only by the direct mixing of the acceptor fluid with the SLM dispersion, but above all by the high ratio of this fluid compared to the small volume of SLM. This is a direct result of trying to maintain *sink* conditions. In the case of the administration of eye drops, such a situation would not occur at the site of application; therefore, the MFS model cannot be considered predictive of real conditions. Moreover, for this method, after the *burst effect*, further release of a significant amount of Cs is usually no longer observed. This means that in a short time, the entire amount of the substance is washed out of SLM without significant degradation of the microparticles.

No *burst effect* was observed in any model with a membrane. Figure 5 presents the results obtained using only a membrane with an MWCO of 1000 KDa. Therefore, the significant differences obtained between the DBDT and DCh methods result from the model and do not depend on the membrane properties. Regardless of the Cs concentration in the SLM in horizontal chambers, no formulation released more than 14% Cs over 96 h of the study. For comparison, 60–70% of Cs was released from the dialysis bag over the same time period. The similar course of the release profiles obtained by the DBDT method also confirms and indicates the lack of a limiting effect of the membrane; otherwise, the SLM profiles with 0.5% Cs and 4% Cs would differ significantly.

The differences between the DBDT and DCh methods may be justified by the design of the Side-Bi-Side chambers used for the DCh method and their volume. Comparing both methods with a membrane, the DBDT technique definitely makes it easier to prepare a sample. Maintaining *sink* conditions is also easier due to the possibility of flexible selection of both the bag volume and the compartment with the acceptor fluid. This allows testing of formulations in a wide range of drug substance concentrations. According to the recommendations, the inside volume of the dialysis bag should be at least 6–10-fold less than that of the outer acceptor fluid in order to provide a driving force for drug transport across the dialysis membrane [25,36]. Another significant difference between the methods using a membrane is its surface area (see Section 2.4). In order to eliminate the surface effect, Figure 5b presents the Cs release results expressed in µg/cm^2^ of the membrane. Although the same membrane is used, Cs permeation is generally slower when using the DCh model than for the DBDT.

The dilution of the SLM dispersion in the bag before testing allows for the introduction and provision of direct contact of the LS with the microspheres. This procedure also imitates the mixing of the SLM dispersion with the tear fluid in the conjunctival sac after application, while avoiding significant dilution that would not occur under physiological conditions. Some concerns have been expressed in other references [25] about ensuring adequate mixing to prevent microparticle aggregation within the dialysis bag. In our study, inhomogeneity of SLM dispersion was observed in the horizontal chambers [23], despite the use of magnetic stirrers in both chambers. Both of the membrane methods also eliminate the need to separate the released substance from the dosage form, making sampling relatively easy and eliminating unwanted carrier loss during sampling.

## 4. Conclusions

Release testing is a key tool for improving and developing dosage forms. In the case of modern, multicompartment lipid carriers, such as SLM, there is a lack of clear pharmacopoeial guidelines or recommendations from registration agencies. Therefore, continuous development and improvement of methods optimally matched to test the properties of the drug carrier is essential.

The dialysis bag method was considered the most appropriate for testing the release of Cs from SLM out of the three compared methods. The key for this method is the selection of a membrane with an appropriate MWCO and the volume of the donor and acceptor compartments. In the studies of SLM with Cs, due to its structure, it was necessary to use a 1000 KDa membrane. As an acceptor fluid, an isotonic sodium chloride solution with the addition of SLS is recommended to ensure appropriate solubility of Cs. The characteristic distribution of Cs in lipid microspheres results in a prolonged release, which, however, is not complete (even in the presence of the lysozyme enzyme) without degradation of the lipid matrix.

Both the results presented in this paper and our previous research can be summarized as follows. The numerous advantages of the dialysis bag method make it suitable for studying multicompartment lipid carriers, including SLM. Establishing clear guidelines for membrane selection in terms of MWCO requires further research comparing the behavior of various sparingly water-soluble substances, taking into account not only their molecular weight but also their spatial structure. Other tools, such as spatial modeling, could also be considered for this purpose.

## Figures and Tables

**Table 1:** Microparticle size and distribution of cyclosporine in SLM dispersions.

Formulation	Particle Size (µm)			API Distribution (%)		
	d_0.5_	d_0.9_	Mean	Aqueous Phase	Interphase	Lipid Matrix
SLM-Cs 0.5%	1.59	6.66	2.86	0.23	48.8	51.0
SLM-Cs 2.0%	1.89	5.59	2.66	0.35	66.7	32.9
SLM-Cs 4.0%	1.90	10.41	3.84	0.28	63.9	35.8

## Data Availability

Data are contained within the article.
